# Non-randomized rapamycin augmentation of chronic self-administered ketamine provides subjective improvement in diverse phenotypes

**DOI:** 10.3389/fpsyt.2026.1910337

**Published:** 2026-08-26

**Authors:** Jon Berner

**Affiliations:** Woodinville Psychiatric Associates, Woodinville, WA, United States

**Keywords:** bipolar disorder, ketamine, mitochondria, pain, rapamycin

## Abstract

**Background:**

Multiple preclinical and clinical reports suggest that rapamycin augmentation may increase the safety and efficacy of ketamine treatment. Population variability in patient biochemical gain-of-function and phenotypic admixtures, however, has slowed initial investigations into patient selection and dose finding.

**Methods:**

A single-center, retrospective chart review identified 101 patients with ketamine use longer than 2 months. Correlational relationships between demographics, biometrics, ketamine use, concurrent medication use, and serum and tissue biomarkers were obtained in an attempt to characterize this group, given that likely severe selection bias limits generalizability. Rapamycin augmentation was attempted in 38 of these patients. Response was defined by patient preference for continuation and asymptotic dosing.

**Results:**

Ketamine dosing (mean 4.4 ± 3.9 mg/kg/day) ranged widely from 0.4 to 20.7 mg/kg/day. Chronic ketamine use correlated with concurrent opioid use and was possibly associated with various tissue indices of mitochondrial activity. Among 38 patients who attempted rapamycin augmentation, 22 reported phenotypic benefit. Average dose was 1.5 ± 0.95 mg. Phenotypic response was highly variable, with improvement in cognition, fatigue, mood, pain, and dysautonomia. Rapamycin response correlated with weight and absence of antipsychotic use.

**Conclusion:**

Rapamycin and ketamine in combination provide subjective benefit in a select, narrow population. The generalizability of these findings cannot be estimated easily. Subsequent randomized trials may consider using modal dosing as an exploratory endpoint and/or biomarkers, which may have greater utility than phenotypic checklists. These biomarkers could reflect ketamine’s action on complex 1 activity in presynaptic glutamatergic terminals and/or joint effects with rapamycin on homeostatic microglia.

## 1 Introduction

Ketamine provides tremendous benefits for many patients. Ketamine use in current practice has very broad phenotypic indications and very diverse dosing protocols. The benefits of ketamine use have been documented in trials for mood disorder (1), treatment-resistant depression (2), pain (3), opioid dependence (4), and post-traumatic stress disorder (PTSD) (5); case reports describing response in psychosis also exist, challenging the academic n-methyl-d-aspartate (NMDA) receptor hypofunction model of schizophrenia (6). Routes of administration vary from weekly intravenous (IV) treatments for mood disorder, to daily IV treatments for pain, as well as self-administration via multiple routes (intranasal [IN], oral [PO], sublingual [SL], intramuscular [IM], and rectal [PR]) and frequencies.

Unfortunately, ketamine alone is not enough for many patients. Age-related progression of primary disease occurs in many despite initial benefit. Some patients are tantalized by a partial but non-curative effect, while others have intolerable side effects despite obvious benefit. Inconvenient or expensive monitored protocols pose additional barriers to successful treatment. The individual patient who has a complicated relationship with ketamine presents a major challenge to clinicians.

The diversity of clinical experience has created a “Tower of Babel” complexity in the academic and clinical literature. Proposed targets for ketamine’s mechanism of action range from NMDA-mediated effects on parvalbumin-positive interneurons and subsequent prefrontal cortex glutamate release (7), to M1 microglia (8–10), and/or to NFATC4 receptors in cortical pyramidal neurons (11), to name a few. Physician-scientists have too often relied on small datasets from specific clinical sites, patient populations, or unique animal models. Within complex biochemical environments, they confidently suggest mechanisms of action, assuming these findings apply broadly to other clinical and research contexts. Accordingly, clinical guidelines for practitioners often use subtle authoritative language regarding indications and dosing; these guidelines likely do not fully capture the Gaussian variability in biochemical processes within larger human populations (12). The failure of these authoritative models is clearly manifest by billions of dollars of failed investments into alternative NMDA receptor antagonists, which have underperformed on efficacy and safety relative to ketamine (13), and/or poor *a priori* clinical identification of ketamine responders based on phenotypic stratification (2).

One medication, rapamycin, holds particular promise as an augmentation agent for ketamine. General safety is highly likely at low dosages, given its known salutary effects on lifespan extension across multiple vertebrate species and improved healthspan in humans (14). Logical mechanistic synergies have been documented in multiple contexts: animal and depressed human cultured monocyte preparations (9, 10), synergistic upregulation of endothelial nitric oxide synthesis in cultured astroglial cells (15), preliminary data regarding synergistic antidepressant effects of these drugs in human populations (7), and modulation of the terminal course of complex 1 loss of function in the Ndufs4 rodent model (16). Concurrent rapamycin use in preclinical studies also appears to mitigate the risk of ketamine cystitis (17).

This retrospective chart review attempts to integrate real-world clinical experience with chronic combined ketamine and rapamycin use with novel mechanistic hypotheses. The study design is exploratory, designed to generate hypotheses rather than definitive conclusions or guidelines regarding the use of these medications.

## 2 Materials and methods

### 2.1 Study design

This study included a retrospective chart review of patients who presented to a fee-for-service solo psychiatric practice in an upper-middle-class suburban community in the United States. Data gathered included demographics, biometrics, simple diagnoses, and current/historical medication use. Patient characteristics are provided in **Supplementary Table 1**. Biometrics in standard clinical use were available for a limited set of patients (**Supplementary Table 2**): fasting blood sugar, triglyceride-to-HDL (high-density lipoprotein) ratio, serum insulin-to-BMI (body mass index) ratio, RDW (red blood cell distribution width), GFR (glomerular filtration rate), neutrophil-to-lymphocyte ratio, and ALT (alanine transaminase).

Remote chart review also revealed 10 individuals who elected to undergo further testing of mitochondrial function using buccal samples. Objective testing was clinically indicated, given substantial residual disease despite palliative treatment with daily ketamine. Of note, all ongoing experimental trials of palliative treatments for mitochondrial disease require a definitive tissue biopsy for enrollment. The cost of the procedure ($200) and the inconvenience in arranging transportation for the frozen specimen clearly limited participation. Adult healthy controls (N = 24) from previous studies conducted by the testing laboratory served as reference norms for biochemical investigations.

### 2.2 Buccal tissue sample collection and analysis

Buccal samples were harvested from each subject using Catch-All Buccal Collection Swabs (Epicenter Biotechnologies, Madison, WI) at a time of the patient’s choosing and subsequently shipped frozen to the Mitochondrial Disease Laboratory at St. Christopher’s Hospital for Children, Drexel University College of Medicine, Department of Pediatrics, Neurology Section, Philadelphia, PA, USA. Buccal extracts were subsequently prepared using an ice-cold buffered solution (Buffer A, ABCAM) containing a membrane-solubilizing non-ionic detergent, a protease inhibitor cocktail, and then cleared of insoluble cellular material by high-speed centrifugation at 4 °C. Duplicate aliquots of the protein extract were analyzed for protein concentration using the bicinchoninic acid method (Pierce Biotechnology, Rockford, IL). Samples were typically stored at -80 °C for up to 1 week prior to enzymatic analysis.

Dipstick immunocapture assays were used to measure the mitochondrial RC-1 activity levels using 50 µg of buccal extract protein as previously described, with all samples assessed in duplicate (18–20). Signals were quantified using a Hamamatsu Immunochromato reader (MS 1000 Dipstick reader, ABCAM). Raw mABS (milliAbsorbance) results were corrected for protein concentration and data were expressed as percentages of the values obtained with control extracts performed on the same test date. Mitochondrial enzyme activity levels of buccal respiratory complex IV (RC-IV) and of the Krebs cycle enzyme citrate synthase (CS) were assessed using standard spectrophotometric procedures in a 0.5 ml reaction volume as described previously (19), and the data shown represent the mean of two determinations for each enzyme assay. Specific activities of respiratory complexes were normalized to buccal CS activity levels, a commonly used gauge of overall mitochondrial content, and expressed as activity ratios (RC-1/CS and RC-IV/CS). The use of activity ratios, rather than absolute specific activities, in mitochondrial enzyme assessment is well established (21) and, in our experience, yields a much narrower range of control values than activities normalized to the buccal sample’s protein content.

### 2.3 Statistical analysis

Associations between variables were assessed in an exploratory correlation matrix using Pearson’s product-moment correlation coefficient, computed as the point-biserial correlation. Correction for multiple comparisons was not carried out. Cases with missing data on any variable in the analysis set were excluded listwise, yielding a single consistent analytic sample size. A significance threshold of α = 0.05 was applied unless otherwise specified. The SPSS software suite (IBM) was used to conduct all statistical analyses.

### 2.4 Informed consent

Retrospective analysis of human data, de-identified and gathered in the standard course of clinical practice, is exempt from IRB review when collated for quality assurance and medical education purposes absent commercial interests. This exemption was applied to the initial 101-patient cohort. Patients identified as having undergone mitochondrial testing provided informed consent retrospectively, signing forms approved by the Western Institutional Review Board (#20141547), given more stringent interpretations of the Common Rule at that time. All control study patients had provided informed consent for their respective assays as part of previously published proof-of-concept studies (19).

## 3 Results

### 3.1 Characteristics of the ketamine cohort

A portion of the current (as of 5/1/2026) patient caseload was maintained on chronic ketamine. A query of the prescription monitoring program (11/14/2025) for “General Anesthetics,” along with a contemporaneous chart review, identified 101 of 2216 unique patients with self-administered ketamine for more than 2 months, representing approximately 4.5% of the total caseload. Route of administration was predominantly oral taken daily, sublingual or intranasal routes or intermittent dosing were converted into daily oral equivalents (22). Prevalence of primary billing diagnoses provided an estimate of referral bias: bipolar 23%, depression 14%, anxiety 13%, pain syndrome 13%, ADHD 12%, psychosis 7.5%, mild cognitive impairment 7%, and autism 6%. Consistent with this referral bias, patients were maintained on typical combinations of antidepressants, antipsychotics, anticonvulsants, lithium, benzodiazepines, psychostimulants, hypnotics, and opioids. The cohort was 61% female and had a mean age of 46.3 ± 14.2 years, ranging from 18 to 87 (**Supplementary Table 1**).

A second, partially intersectional remote chart review conducted for the years 2013–2014 identified 10 patients, predominantly female (7 F/3 M), who elected to undergo mitochondrial function testing and were analyzed in greater detail. Mean age was 50 ± 13.4 years, ranging from 30 to 75. Clinical indications were refractory pain (N = 7) and refractory depression (N = 3). Six patients had symptoms suggestive of mitochondrial dysfunction. The mean age of the control group was 44 years and comprised 15 females and 9 males (**Table 1**) (19).

**Table 1:** Patient characteristics of mitochondrial function cohort.

Patient	Age	Gender	Ketamine dose (mg/kg)	Ketamine duration (months)
1	47	Female	11.7	67
2	48	Female	14.2	79
3	50	Female	8.7	30
4	64	Female	16	36
5	48	Male	23	45
6	36	Female	20	51
7	44	Female	5.7	81
8	75	Male	2	51
9	62	Female	17	30
10	30	Male	8.4	43
Patients, mean (SD)	50.4 (13.4)	3 M/7 F	12.7 (6.6)	51.3 (18.7)
Controls, mean (SD)	43.6 (13.8)	9 M/15 F	N/A	N/A

**Figure 1:**
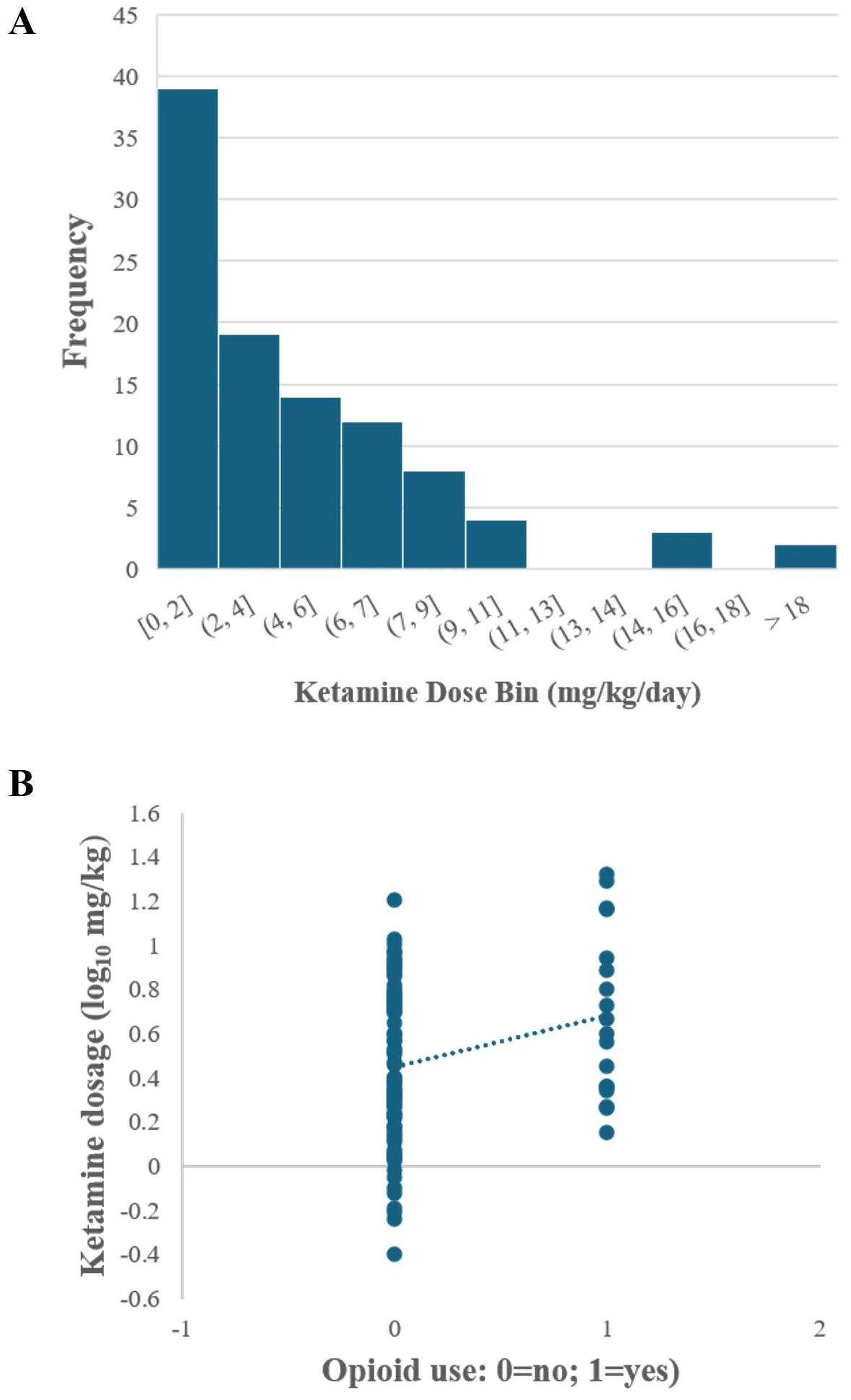
Distribution of ketamine dose and correlation with opioid use. **(A)** Histogram demonstrates the range and skewness of ketamine doses self-administered daily within the 101-patient cohort. **(B)** The relationship between ketamine dose and opioid use in the 101-patient cohort. Pearson Correlation: r=0.245; 2-tailed significance = 0.014; 95% CI: 0.052 to 0.421.

Ketamine dosing had a large coefficient of variation (mean 330.1 ± 263.2 mg) with a range of 32 to 1350 mg daily (**Figure 1A**). The mean dose of ketamine in oral equivalents was 4.39 ± 3.92 mg/kg, ranging from 0.4 to 20.7 mg/kg/day. Time on drug averaged 42 months, ranging from 2 to 204 months (**Supplementary Table 2**). Given the extent of skewness in the dose distribution, log_10_ (ketamine mg/kg) was correlated with clinical variables. For the complete exploratory correlation matrix see **Supplementary Table 3**. Higher doses of ketamine were uniquely correlated with opioid use (r=0.245; p=0.014; 95% CI: 0.052 to 0.421) (**Figure 1B**).

In the mitochondrial function cohort, the mean duration of ketamine use was 51.3 ± 18.7 months with a range of 30 to 81 months (**Table 1**). Route of administration varied between PO (n=4), IN (n=4), PR (n=1), and IM (n=1).

### 3.2 Biochemical changes associated with chronic ketamine use, distinct from behavioral phenotype

Serum markers of biological responses to ketamine use were collected in a subset of patients (**Supplementary Table 2**). Ketamine use per se was not associated with markers of metabolic stress (fasting blood sugar, triglyceride/HDL ratio, serum insulin/BMI ratio), hepatic stress (ALT), bone marrow stem cell composition (RDW, neutrophil/lymphocyte ratio), kidney function (GFR), clinical characteristics (age, gender, height, weight), or other psychotropic drug use, suggesting that chronic low-dose ketamine use has a benign toxicity profile. Ketamine dosage trended towards a positive association with ALT elevations; however, a single outlier corresponding to chronic EBV infection accounted for most of the variance in the full dataset (**Supplementary Figure 1A**) vs. the annotated dataset (**Supplementary Figure 1B**).

Ketamine was strongly associated with changes in complex 1 activity in a buccal biopsy. Although the mean total protein (666 ± 153 μg) and RC-IV activity/CS activity ratio (0.25 ± 0.14) in the ketamine group were similar to the control population, the RC-1/CS activity ratio was reduced, and citrate synthase activity was elevated (**Table 2**). In 7/10 individuals, RC-1/CS significantly differed from the 95% confidence interval of the control distribution, with average levels in the group (2.86 ± 2.78) significantly lower than the control group (6.7 ± 2.0) (t= -4.59, p=0.00). While 1/10 individuals had citrate synthase activity levels significantly different from the 95% control confidence interval, mean levels in the ketamine group were 26.6 ± 12.4 nmoles/min/mg, which was significantly higher (t=2.91, p=0.02) than the control group (12.1 ± 5.5 nmoles/min/mg). One patient discontinued ketamine for 3 weeks to test the hypothesis that ketamine, rather than patient selection, accounted for observed abnormalities. RC-1/CS activity ratio increased from 3.7 to 8.6, a low normal to high normal range, and represented a statistically greater than 2 SD increase. In contrast, this patient displayed no significant change in buccal CS or RC-IV/CS activity.

**Table 2:** Ketamine effects on complex 1 activity.

Patient		Buccal protein yield, μg (%)^	CS (citrate synthase) nmoles/min/mg (%)^	CI/CS (complex I) ratio (%)^	CIV/CS (complex IV) ratio (%)^	Mitochondrial studies
1		440 (68%)	22.2 (182%)	1 (15%)*	0.14 (42%)	+serum alanine
2		543 (88%)	28.4 (23%4)	2.8 (41%)*	0.17 (52%)	+glutaric acidemia, low serum BAA
3		885 (144%)	29.8 (246%)	1.1 (16%)*	0.16 (48%)	+C2-C5 acylcarnitines, +glutamic acid cysteine serum-nextgen sequencing
4		748 (122%)	25 (206%)	9.3 (139%)	0.52 (158%)	
5		900 (146%)	60 (495%)*	2 (29%)*	0.15 (47%)	Multiple buccal mtDNA heteroplasmies
6		680 (110%)	18 (149%)	<1 (<15%)*	0.14 (44%)	RYR2 nextgen Low BAA High glycine
7		472 (77%)	21.5 (17%7)	1.2 (18%)*	0.32 (100%)	+serum alanine
8		594 (97%)	24.2 (5%0)	5.7 (85%)	0.2 (61%)	C5-C18 acylcarnitines
9		648 (105%)	17.3 (14%3)	1.8 (27%)*	0.24 (73%)	
10	*Test 1*	*520 (85%)*	*19 (157%)*	*3.7(55%)*	*0.46 (139%)*	No significant deficiencies
	*Test 2§*	*990 (161%)§*	*19.4 (159%)§*	*8.6 (128%)§*	*0.46 (139%)§*	
	Mean	755 (122%)~	19.2 (158%)	6.15 (92%)	0.46 (139%)	
Patients, mean (SD)		666 (108)	26.6 (12.5)	3.2 (2.9)	0.25 (0.14)	
Controls, mean (SD)		615	12.1 (5.5)	6.7 (2.0)	0.33 (0.1)	

^Percentage of Control mean values.

*Greater than two standard deviations from the control mean.

^§^Retest values after the patient discontinued ketamine use for 3 weeks.

### 3.3 Rapamycin augmentation

Rapamycin augmentation in 38 of the 101 patients provided an estimated 57.9% (95% CI: 42.0%–73.8%) probability of response, with 22 patients reporting a positive phenotypic response. The average dose was 1.5 ± 0.95 mg with a range of 0.5 to 4 mg daily. Duration of use ranged from 1 to 70 months, with a mean of 17.8 months. Phenotypic response was highly variable, with improvement in cognition, fatigue, depression, catastrophic reactions (emotional incontinence seen in autism and frontal lobe injury), pain with infrequent opioid discontinuation without withdrawals, and dysautonomia. Rapamycin response correlated with lack of antipsychotic use (r= -0.381, p=0.018, [95% CI: -0.625, -0.070]), lack of hypnotic use (r=-0.413, p=0.010) [95% CI: -0.647, -0.108]), and body weight (r=0.401, p=0.013, [95% CI: 0.093, 0.639]), also trending to male gender (r=0.277, p=0.092, [95% CI: -0.047, 0.548]) (**Table 3**).

**Table 3:** Correlation of rapamycin phenotypic response to antipsychotic and hypnotic use.

Pearson correlation (2-tailed sig.) [95% CI]	Antipsychotic use N=38	Hypnotic use N=38	Weight N=38	Gender N=38
Probability of rapamycin response	-0.381 (0.018) [-0.625, -0.070]	-0.413 (0.010) [-0.647, -0.108]	0.401 (0.013) [0.093, 0.639]	0.277 (0.092) [-0.047, 0.548]

## 4 Discussion

This retrospective chart review provides proof of concept that chronic use of ketamine with rapamycin augmentation provides subjective benefit to a subset of patients, a replicated extension of an acute, time-limited, pilot randomized trial (7). Careful analysis of modal dosing and phenotypic/biomarker admixtures may hopefully provide guardrails for more granular and robust prospective studies.

The observed association of higher dosages of ketamine with opioid use is reported, despite absent randomization, given recent data in rodents that antinociception dosing is higher and physiologically distinct from depression treatment protocols (13). Converging human evidence from gene set enrichment in peripheral blood mononuclear cells (PBMCs) (23), blood phosphorylation (8), and blood monocyte phenotypes (9, 24) documents that ketamine and rapamycin shift the distribution of myeloid species in autoinflammatory disease from a “painful” inflammatory phenotype to the homeostatic phenotype. The suggestion that a ketamine response implies subsequent treatment with low-dose buprenorphine as a sustained prophylactic treatment for suicidal behavior, as observed in a randomized trial, is consistent with these findings (25). However, because a correction for multiple comparisons was not performed due to the uncontrolled and heterogeneous nature of the study cohort, this study should be viewed as exploratory and interpreted appropriately. Further investigation of the relationship between high-dose ketamine and opioid use is warranted and highly relevant.

Selection bias in this study is massive. Patients are recruited from the 18th-richest city in the world. Patients willing to engage in novel treatments are often more educated than a random sample and can cover the costs of compounded medications or uncompensated assays. Genome-wide association analysis (26) and tissue biopsy (12) clearly show that these behavioral phenotypes are not solely environmentally determined and have strong effects on metabolic health, inflammatory disease, depression subtypes, and alcoholism. Patient preference for augmentation is also strongly driven by disease severity; progressive disease, absent nihilistic depression, drives risk-taking. The inverse possibility that subtle bipolar mania, “flight into health”, based on longevity advertisements, is also possible. Provider selection bias is also likely present, as an undefined clinical “intuition” about likely treatment responders evolved over time, coinciding with the rapid expansion of the clinical literature on low-dose rapamycin.

Another major limitation of this retrospective review is the low information density of the phenotypic description of patients. The range and magnitude of the phenotypic response to non-randomized treatment cannot practically be derived from retrospective clinical chart review. Patients in a fee-for-service practice have minimal interest in rating scales commonly used in academic settings, preferring unstructured narratives, which may actually be more in line with current DSM proposed revisions. Recent studies show that dimensional checklists “find no zones of rarity among patient clusters.” In other words, patients are distributed smoothly from low to high numbers on all dimensions without leaving clear gaps between clusters of patients” (27). In the context of frequent complicated phenotypic admixtures observed in this population, illustrated by medication and diagnostic overlaps in the cohort, it may be necessary in the future to design prospective trials differently. Biomarkers with greater information density than patient self-report, clinician assessment, biometrics, or standard laboratory work could potentially simplify ketamine and rapamycin dose-finding. We note again, as replicated in this study, that even with multiple markers and associated statistical fragility, standard low-cost clinical phenotypes had minimal predictive validity for patient selection and dose finding (2).

Although in this study buccal respirometry superficially appears to document chronic effects of ketamine on mitochondrial quality control and has recently been used for the management of autism spectrum disorder (28), without prospective studies controlling for referral bias, its utility remains uncertain. Further adjunctive use of the Bispectral Index EEG, commonly used to guide anesthesia dosing (29) or serum markers of autophagy, e.g., pATG13 and serum CD206 density predictive of rapamycin response in post-viral chronic fatigue (24, 30), could be considered.

Further translational work with ketamine might revisit its historical role as an anesthetic at higher dosages, inconsistent with pure NMDA antagonists like memantine and MK-801; then attention shifts to its preferential target, the presynaptic glutamatergic terminals. These terminals are highly energy-intensive, requiring large ATP fluxes to recycle lipid vesicles used during endocytosis (31). All anesthetics appear to bind complex 1 in the mitochondria and disrupt the electron transport chain locally, leading to a reduction in ATP availability (32) and acute unconsciousness, absent sustained glutamatergic signaling. Low chronic doses of anesthetic, even without impaired consciousness, can produce subsequent mitochondrial membrane depolarization, altered coupling, and electron leakage at the Fe-S clusters in complexes 1 or III, affecting homeostatic production of reactive oxygen species. Disruptions in electron flow produce downstream effects on presynaptic mitochondrial quality control, the mitochondrial unfolded protein response, and related local signals associated with microglial efferocytosis of damaged synapses. These mitochondrial-disruptive and variably toxic-to-beneficial effects of ketamine depend on the specific agent, dosage, tissue context, and exposure duration (33).

Paradoxically, modest inhibition of complex 1 may actually lower rates of dementia and/or mortality, as has recently been documented in animals with transcriptomic signatures of Alzheimer’s dementia (Ndufs 4 null) (34). Although the animals cannot provide feedback to the researchers regarding the subjective effects of these agents, depressed and anxious patients exposed to inhalational anesthetics other than ketamine, such as isoflurane (4) and xenon (35), report sustained mood improvement subsequent to their acute anesthetic experience. Note that xenon, as a noble gas with completely filled valence electron shells, is largely chemically inert; its proximal activity is depolarization of the electron flux through the multiple subunits of complex 1, not chemical modulation of NMDA receptor (32). Accordingly, agents that activate NRF2 and/or mitochondrial turnover, as observed subsequent to C1 inhibition in animal models (34), could also potentially serve as additional logical augmentation agents as distinct from this index trial with rapamycin.

### 4.1 Conclusions

Rapamycin and ketamine in combination provide subjective benefit in a select, narrow population. The generalizability of these findings cannot be estimated easily. Subsequent randomized trials may consider using modal dosing as an exploratory endpoint and/or biomarkers, which may have greater utility than phenotypic checklists. These biomarkers could reflect ketamine’s action on complex 1 activity in presynaptic glutamatergic terminals and/or joint effects with rapamycin on homeostatic microglia.

## Data Availability

The raw data supporting the conclusions of this article will be made available by the authors, without undue reservation.
