# Supplementary material for: Non-randomized rapamycin augmentation of chronic self-administered ketamine provides subjective improvement in diverse phenotypes
Source: Front Psychiatry. 2026 Aug 26;17:1910337. doi: 10.3389/fpsyt.2026.1910337 (PMC13553960; doi:10.3389/fpsyt.2026.1910337)
Supplement: Supplementary file 1 [file SupplementaryFile1.pdf]

Supplementary Material

1 Supplementary Tables

1.1 Supplemental Table 1: Chart review patient characteristics

	N	Mean	SD	%
Age (years)	101	46.36	14.17	
Female gender	101			61.4
Height (inches)	101	67.40	4.02	
Weight (lbs)	101	178.90	46.77	
Weight (kg)	101	81.32	21.26	
BMI (kg/m^2)	101	27.41	6.68	
Ketamine Use				
Ketamine daily dose (mg)	101	330.09	263.17	
Ketamine dose (mg/kg)	101	4.39	3.92	
Ketamine duration if use (months)	101	42.41	43.77	
Rapamycin Use				
Rapamycin use	101			21.8
Rapamycin dose (mg/day)	22	1.50	0.93	
Rapamycin duration of use (months)	22	17.77	19.97	
Other Drug Use				
Pain: presence of pain diagnosis	101			66.3
Mood: depression, anxiety or bipolar diagnosis	101			77.2
Synapse: schizophrenia or autism diagnosis	101			11.9
IV ketamine: previously tried IV ketamine	101			29.7
Antidepressant: using an antidepressant	101			36.6
DA2: using an antipsychotic	101			24.8
AED: using an anticonvulsant	101			45.5
Lithium: using lithium	101			11.9
Benzodiazepine: using benzodiazepine	101			44.6
Stimulant: using psychostimulant	101			42.6

Hypnotic: using z-drug, a1 agonist or sedating antidepressant	101			30.7
Opioid: using full agonist or buprenorphine	101			17.8

Supplemental Table 2: Serum drug safety markers

	N	Mean	SD	Normal Range
Fasting Blood Glucose (mg/dL)	35	92.80	9.77	70-99
TG:HDL Ratio	35	3.07	2.60	<3
Insulin:BMI Ratio	25	2.29	1.56	0.1-1.35
RDW (%)	28	12.63	2.51	11.5-14.5
GFR (mL/min/1.73 m^2)	37	90.92	17.57	≥90
Neutrophil:Lymphocyte Ratio	29	2.04	0.98	1-3
ALT (U/L)	21	33.81	34.37	7-55

Abbreviations: ALT, alanine transaminase; BMI, body mass index; GFR, glomerular filtration rate; HDL, high-density lipoprotein; RDW, red blood cell distribution width; TG, triglycerides.

Supplemental Table 3: Exploratory Pearson Correlation Matrix

Variable	Rapa Response	Gender	Age	Height	Weight	BMI	Ket Dose	Log10 Ket Dose	Ket Weight	Anti-depressant Use	DA2 (Anti-psychotic) Use	Anti-convulsant Use	Lithium Use	Benzo-diazepine Use	Psycho-stimulant Use	Hypnotic Medication Use.	Opioid Use
**Rapamycin Response**	1.000	.277 (.092) *[38]*	-.183 (.272) *[38]*	.224 (.177) *[38]*	**.401 (.013)** *[38]*	.310 (.058) *[38]*	-.111 (.505) *[38]*	-.270 (.101) *[38]*	-.234 (.158) *[38]*	-.063 (.706) *[38]*	**-.381 (.018)** *[38]*	.139 (.407) *[38]*	-.119 (.475) *[38]*	-.231 (.162) *[38]*	.064 (.701) *[38]*	**-.413 (.010)** *[38]*	-.191 (.251) *[38]*
**Gender**	.277 (.092) *[38]*	1.000	.063 (.530)	**.671 (.000)**	**.381 (.000)**	.089 (.376)	.071 (.482)	-.067 (.506)	-.067 (.503)	-.181 (.070)	-.125 (.213)	**.214 (.032)**	.149 (.138)	.066 (.509)	-.066 (.512)	.045 (.652)	-.104 (.302)
**Age**	-.183 (.272) *[38]*	.063 (.530)	1.000	.034 (.739)	.095 (.344)	.075 (.456)	.036 (.718)	-.040 (.690)	.015 (.883)	.075 (.455)	-.110 (.274)	.056 (.581)	.060 (.552)	-.044 (.665)	**-.201 (.044)**	.107 (.285)	**.218 (.028)**
**Height**	.224 (.177) *[38]*	**.671 (.000)**	.034 (.739)	1.000	**.377 (.000)**	-.034 (.739)	-.044 (.663)	-.171 (.087)	-.149 (.138)	-.090 (.369)	.023 (.816)	**.217 (.029)**	.131 (.190)	.060 (.548)	-.060 (.551)	.063 (.533)	-.039 (.695)
**Weight**	**.401 (.013)** *[38]*	**.381 (.000)**	.095 (.344)	**.377 (.000)**	1.000	**.894 (.000)**	-.048 (.636)	**-.324 (.001)**	**-.337 (.001)**	-.021 (.833)	.083 (.411)	-.060 (.550)	.042 (.677)	-.077 (.445)	-.041 (.681)	-.007 (.946)	-.001 (.990)
**BMI**	.310 (.058) *[38]*	.089 (.376)	.075 (.456)	-.034 (.739)	**.894 (.000)**	1.000	-.018 (.857)	**-.258 (.009)**	**-.284 (.004)**	.022 (.827)	.101 (.313)	-.154 (.123)	.002 (.985)	-.113 (.262)	-.019 (.850)	-.049 (.624)	.009 (.925)
**Ketamine Dose**	-.111 (.505) *[38]*	.071 (.482)	.036 (.718)	-.044 (.663)	-.048 (.636)	-.018 (.857)	1.000	**.868 (.000)**	**.927 (.000)**	.071 (.480)	-.062 (.536)	-.108 (.285)	-.111 (.267)	.073 (.467)	.073 (.468)	.102 (.308)	**.261 (.008)**
**Log10 Ketamine Dose**	-.270 (.101) *[38]*	-.067 (.506)	-.040 (.690)	-.171 (.087)	**-.324 (.001)**	**-.258 (.009)**	**.868 (.000)**	1.000	**.892 (.000)**	.026 (.794)	-.088 (.384)	-.060 (.551)	-.184 (.065)	.134 (.183)	.023 (.817)	.077 (.443)	**.245 (.014)**
**Ketamine Weight**	-.234 (.158) *[38]*	-.067 (.503)	.015 (.883)	-.149 (.138)	**-.337 (.001)**	**-.284 (.004)**	**.927 (.000)**	**.892 (.000)**	1.000	.076 (.451)	-.080 (.429)	-.091 (.366)	-.118 (.240)	.097 (.335)	.061 (.547)	.140 (.162)	**.303 (.002)**
**Anti-depressant Use**	-.063 (.706) *[38]*	-.181 (.070)	.075 (.455)	-.090 (.369)	-.021 (.833)	.022 (.827)	.071 (.480)	.026 (.794)	.076 (.451)	1.000	.088 (.383)	-.035 (.727)	-.152 (.129)	.021 (.833)	.010 (.919)	.029 (.776)	.129 (.198)
**DA2 (Anti-psychotic) Use**	**-.381 (.018)** *[38]*	-.125 (.213)	-.110 (.274)	.023 (.816)	.083 (.411)	.101 (.313)	-.062 (.536)	-.088 (.384)	-.080 (.429)	.088 (.383)	1.000	.166 (.096)	**.215 (.031)**	.086 (.393)	-.030 (.767)	.116 (.249)	.093 (.357)
**Anti-convulsant Use**	.139 (.407) *[38]*	**.214 (.032)**	.056 (.581)	**.217 (.029)**	-.060 (.550)	-.154 (.123)	-.108 (.285)	-.060 (.551)	-.091 (.366)	-.035 (.727)	.166 (.096)	1.000	.094 (.348)	.100 (.319)	-.104 (.301)	.038 (.706)	-.114 (.256)
**Lithium Use**	-.119 (.475) *[38]*	.149 (.138)	.060 (.552)	.131 (.190)	.042 (.677)	.002 (.985)	-.111 (.267)	-.184 (.065)	-.118 (.240)	-.152 (.129)	**.215 (.031)**	.094 (.348)	1.000	.040 (.690)	-.131 (.193)	.087 (.385)	-.091 (.365)

Variable	Rapa Response	Gender	Age	Height	Weight	BMI	Ket Dose	Log10 Ket Dose	Ket Weight	Anti-depressant Use	DA2 (Anti-psychotic) Use	Anti-convulsant Use	Lithium Use	Benzo-diazepine Use	Psycho-stimulant Use	Hypnotic Medication Use.	Opioid Use
**Benzo-diazepine Use**	-.231 (.162) *[38]*	.066 (.509)	-.044 (.665)	.060 (.548)	-.077 (.445)	-.113 (.262)	.073 (.467)	.134 (.183)	.097 (.335)	.021 (.833)	.086 (.393)	.100 (.319)	.040 (.690)	1.000	.155 (.122)	.181 (.070)	.103 (.305)
**Psycho-stimulant Use**	.064 (.701) *[38]*	-.066 (.512)	**-.201 (.044)**	-.060 (.551)	-.041 (.681)	-.019 (.850)	.073 (.468)	.023 (.817)	.061 (.547)	.010 (.919)	-.030 (.767)	-.104 (.301)	-.131 (.193)	.155 (.122)	1.000	.035 (.730)	-.087 (.387)
**Hypnotic Medication Use**	**-.413 (.010)** *[38]*	.045 (.652)	.107 (.285)	.063 (.533)	-.007 (.946)	-.049 (.624)	.102 (.308)	.077 (.443)	.140 (.162)	.029 (.776)	.116 (.249)	.038 (.706)	.087 (.385)	.181 (.070)	.035 (.730)	1.000	.195 (.051)
**Opioid Use**	-.191 (.251) *[38]*	-.104 (.302)	**.218 (.028)**	-.039 (.695)	-.001 (.990)	.009 (.925)	**.261 (.008)**	**.245 (.014)**	**.303 (.002)**	.129 (.198)	.093 (.357)	-.114 (.256)	-.091 (.365)	.103 (.305)	-.087 (.387)	.195 (.051)	1.000

Values shown are Pearson r with two-tailed p-value in parentheses. **Bold red** values indicate $p < .05$. Rapamycin response correlations are based on N = 38; all other correlations N = 101 (shown in brackets when N = 38). Diagonal entries (1.000) are shown in grey.

2 Supplementary Figures

2.1 Supplemental Figure 1: Correlation of ketamine use with liver function

A

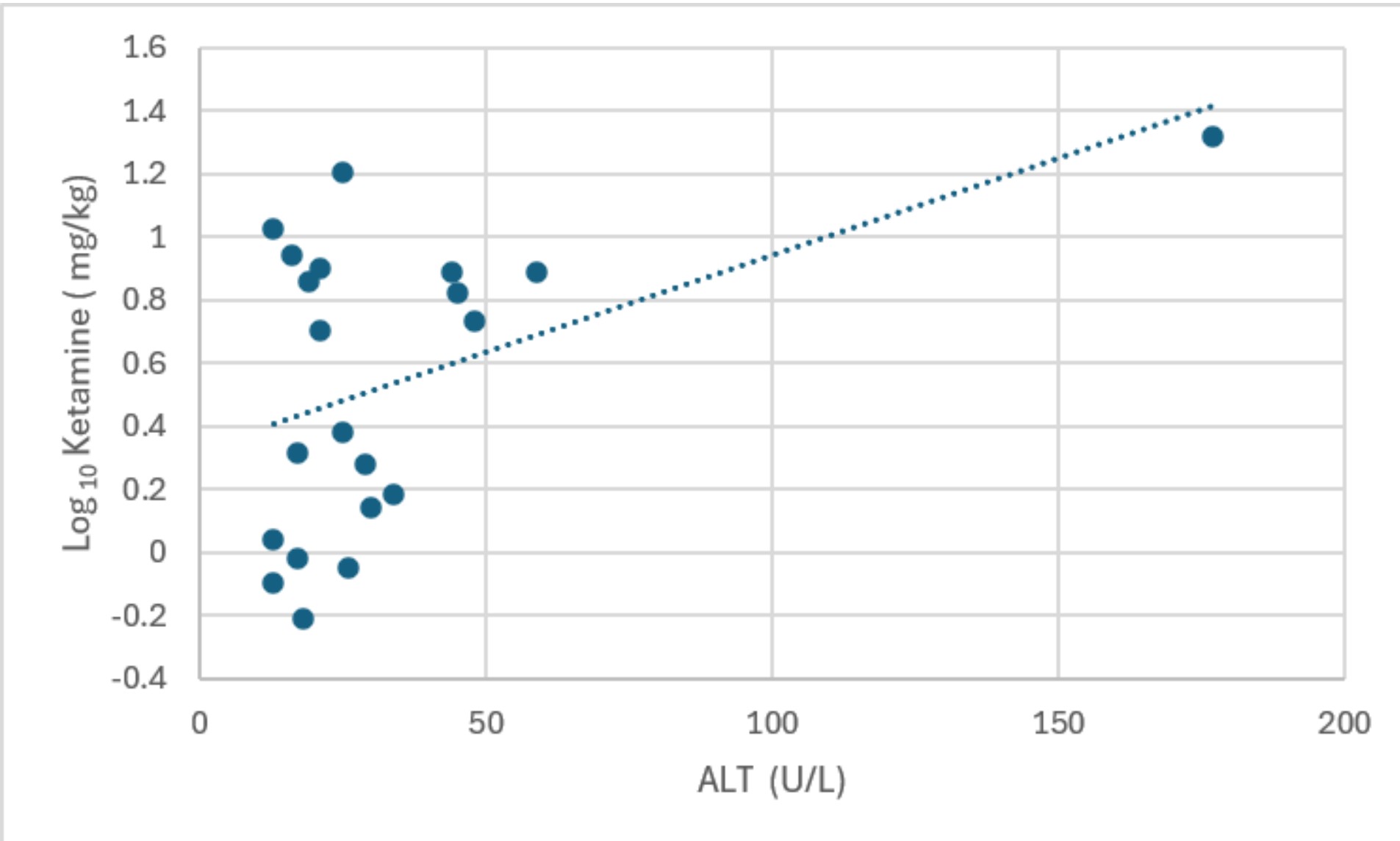

Relationship between ketamine dose and alanine transaminase (ALT) in the full 101 patient cohort. Pearson Correlation (with outlier): r=0.464; 2-tailed significance = 0.034; 95% CI: 0.040 to 0.746

B

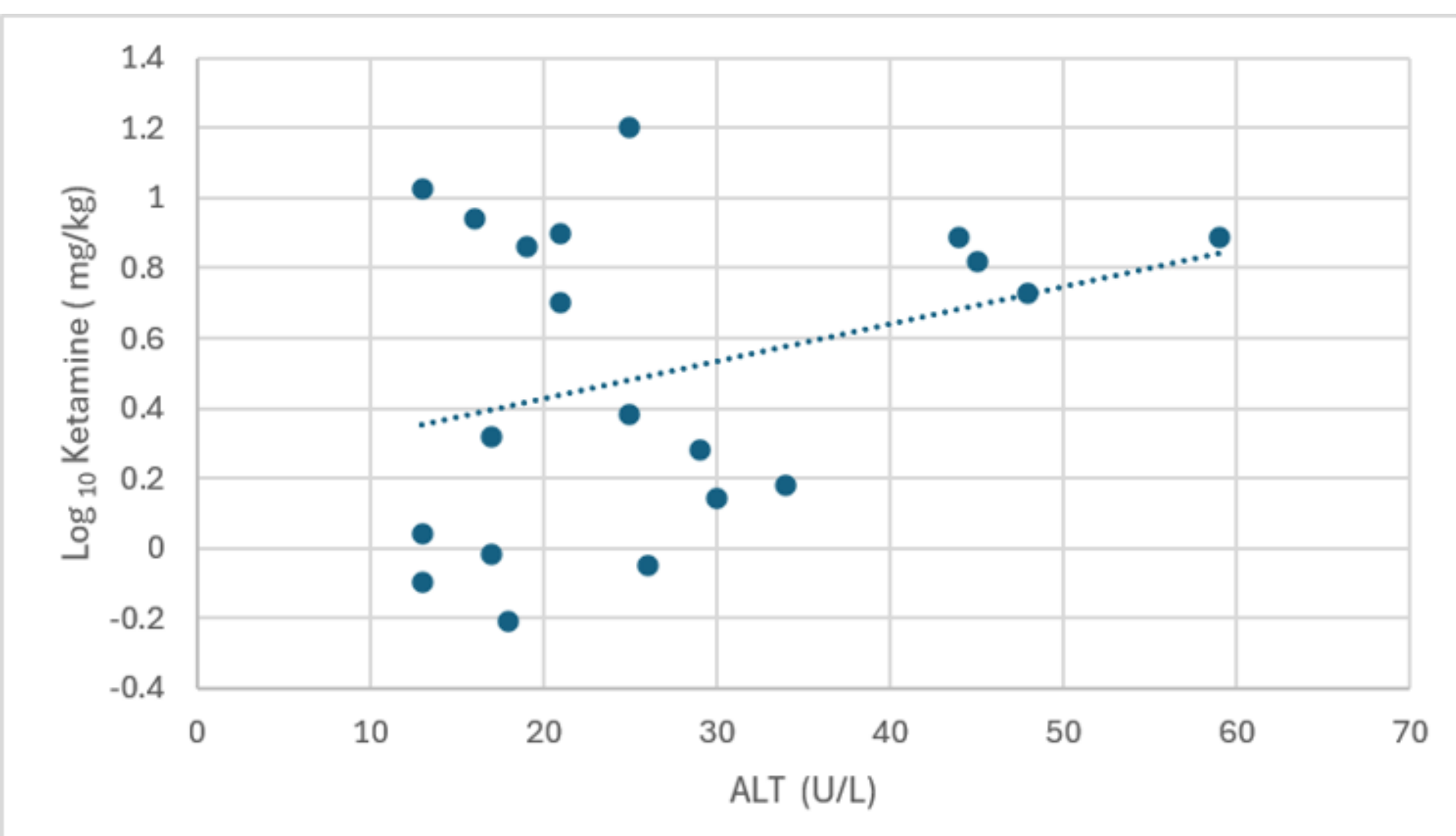

Relationship between ketamine dose and alanine transaminase (ALT) with the single outlier harboring a chronic EBV infection removed. Pearson Correlation: r=0.315; 2-tailed significance = 0.175; 95% CI: -0.148 to 0.665
